# A national survey of the provision of prehabilitation for oesophagogastric cancer patients in the UK

**DOI:** 10.1308/rcsann.2024.0092

**Published:** 2024-11-21

**Authors:** S Barman, RC Walker, PP Pucher, S Jack, G Whyte, MPW Grocott, M West, N Maynard, T Underwood, J Gossage, A Davies, On behalf of the OG Prehabilitation Group and AUGIS (Association of Upper GI Surgeons)

**Affiliations:** ^1^ Guy’s and St Thomas’ NHS Foundation Trust, UK; ^2^ King’s College London, UK; ^3^ University of Southampton, UK; ^4^ Portsmouth Hospitals University NHS Trust, UK; ^5^ University Hospital Southampton NHS Foundation Trust, UK; ^6^ Liverpool John Moores University, UK; ^7^ Oxford University Hospitals NHS Foundation Trust, UK; ^8^ University of Oxford, UK

**Keywords:** Prehabilitation, Oesophagogastric cancer, OGC centres, Survey

## Abstract

**Introduction:**

Studies have demonstrated that prehabilitation in oesophagogastric cancer (OGC) improves body composition, physical fitness and quality of life, and can reduce surgical complications. However, it is not offered in all OGC centres. Furthermore, definitions, funding and access to services vary. We conducted a survey of prehabilitation in OGC centres in England and Wales.

**Methods:**

OGC centres were identified through the National Oesophago-Gastric Cancer Audit (NOGCA). Survey questions were developed, piloted in two institutions and distributed via email in October 2022. Reminder emails were sent over two months until the survey closed in December 2022.

**Results:**

Responses were received from 28 of 36 centres. There was near-universal agreement that prehabilitation should be considered standard of care for patients on curative pathways (27/28; 96%). Most centres (21/28; 75%) offered prehabilitation. The majority of respondents believed that prehabilitation should commence at diagnosis (27/28; 96%) and consist of at least aerobic training and dietitian input. Most (26/28; 93%) believed access to clinical psychologists should be included; however, only 12 (43%) had access to clinical psychologists. Respondents believed prehabilitation improves quality of life (26/28; 93%), fitness (26/28; 93%), smoking cessation (28/28; 100%), surgical complication rates (25/28; 89.3%), likelihood of proceeding to surgery (25/28; 89.3%) and overall survival (20/28; 71.4%).

**Conclusions:**

Despite barriers to funding and a lack of best practice guidelines, most units deliver prehabilitation. Units require higher quality evidence, consensus on the most important aspects of the intervention and core outcome sets to support the delivery of services and facilitate audit to assess the impact of their introduction.

## Introduction

There are numerous definitions of prehabilitation. For the purposes of this project, it was defined as: “Physical activity and complimentary therapeutic interventions delivered between diagnosis and the end of conventional treatment, to prepare for, manage and reduce the impact of a cancer diagnosis and its treatment”.^1–3^ Prehabilitation incorporates the triad of screening, assessment and intervention to identify those in need of assessment, assess individual needs and deliver personalised interventions.^4,5^ Prehabilitation interventions can be specific to the individual, specific to the disease or applicable to both. Alongside physical activity, current prehabilitation interventions often incorporate nutrition and psychology components, as well as smoking and alcohol cessation to act together to improve outcomes.^6^

Multiple studies have shown prehabilitation to be safe during neoadjuvant treatment and prior to surgery for oesophagogastric (OG) cancer.^7–9^ These studies have demonstrated the potential of prehabilitation to improve body composition, sarcopenia, fitness, reduce surgical complications and improve quality of life.^7–9^ Prehabilitation services, however, are not yet offered throughout all OGC centres in the United Kingdom (UK). Where prehabilitation is delivered, the nature of the intervention, delivery and funding is highly variable. The extended treatment pathway for OGC patients represents an opportunity to deliver effective, evidence-based prehabilitation from the date of diagnosis.^10,11^ To better understand attitudes, provision and challenges to prehabilitation practice in OGC centres in England and Wales a nationwide survey was performed.

## Methods

OGC centres in England and Wales were identified through the National Oesophago-Gastric Cancer Audit (NOGCA).^12^ Google forms were used to iteratively develop survey questions, piloted in two institutions and approved by the co-authors prior to distribution via email in October 2022. From the NOGCA database, the nominated representative for each centre was identified and survey questions were distributed. Reminder emails were sent over two months from September to December 2022 until the survey was closed on 5 December 2022. The survey is presented in Appendix 1 (available online).

Respondents were asked questions related to their current prehabilitation provision, barriers to setting up or maintaining the service and what aspects they felt would be important to an ideal service.

Interventions that might be better described as ‘rehabilitation’, such as those delivered after adjuvant chemotherapy or primary surgery and designed to accelerate and improve recovery, were excluded. Descriptive statistics were used to present number (*n*) and percentage (%) of respondents to each question.

## Results

Contact details for 36 of 37 centres were obtained and responses from 28 centres were received. This represented a 78% response rate among OGC units in England and Wales, with responding centres responsible for ∼86% of the national case load in oesophagogastric surgery.

### Current prehabilitation provision

**Figure 1:**
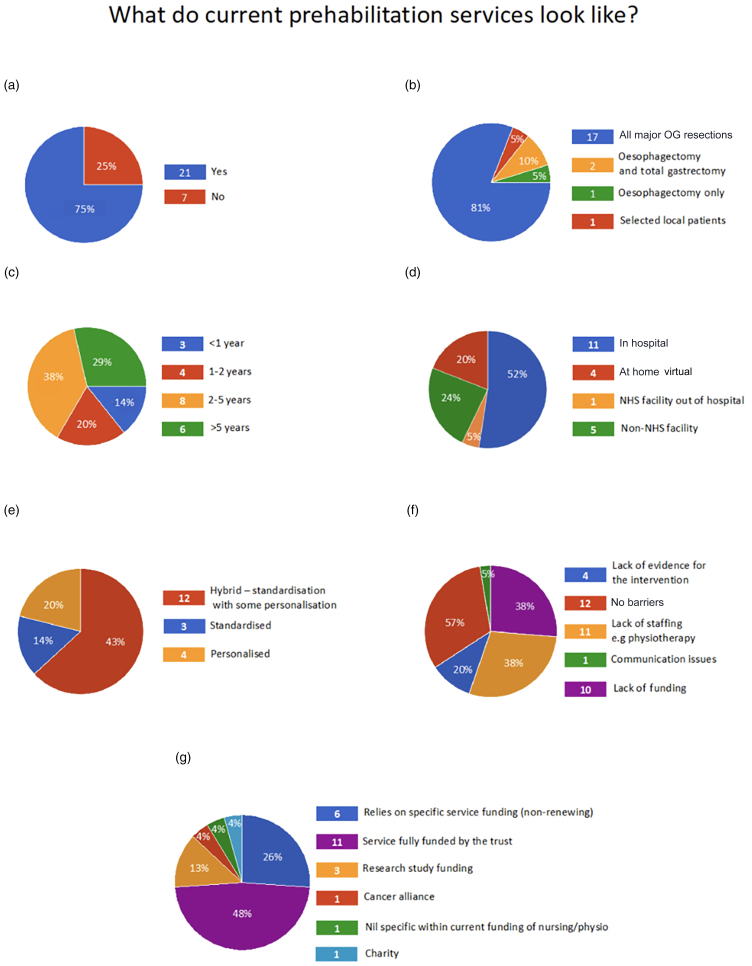
What do current prehabilitation services look like? (a) Pie chart showing the number of respondents already running prehabilitation services. (b) Who is being offered prehabilitation? (c) How long prehabilitation has been offered. (d) Where current services are delivered. (e) Personalisation of services. (f) Barriers to running prehabilitation services. (g) Funding for prehabilitation services.

Respondents were asked if they currently provided prehabilitation services. The majority, 21 of 28 (75%) units, already offered prehabilitation programmes to OGC patients (Figure 1a) and a further 6 units intended to launch them in the future. Of those six units, five had been unsuccessful in launching a programme previously, suggesting ongoing systemic and organisational barriers.

In 17 of 21 centres (81%) prehabilitation was offered to all patients undergoing major OGC resections (Figure 1b) including subtotal gastrectomies. In two centres this was offered to oesophagectomy and total gastrectomy patients only, and in one centre to oesophagectomy patients only. One centre reported variable provision depending on the catchment area. It was not clear whether this variability was due to access to funding or prioritising the highest risk procedures with limited resources. One centre provided a prehabilitation programme for all stages of disease, including during palliative treatment. In three centres the service had been running for less than 1 year, whereas in six centres it had been running for more than 5 years (Figure 1c).

Although most prehabilitation was delivered in hospital (11/21; 52.3%), four units delivered prehabilitation at home virtually (4/21; 19%) and five units in non-National Health Service (NHS) facilities delivered prehabilitation out of hospital (5/21; 23.8%) (Figure 1d). Three units (14.3%) delivered a standardised programme, whereas six units (28.6%) provided a wholly personalised prehabilitation programme. The remaining 12 centres (57.1%) used a hybrid model with some elements standardised and others bespoke to the patient (Figure 1e).

Seventeen centres (81%) had experienced barriers to setting up and maintaining their existing service including: lack of funding (*n* = 10; 47.6%), lack of manpower (*n* = 10; 47.6%) and a perceived lack of evidence for the intervention (*n* = 4; 19%). Of the six units intending to launch prehabilitation services in the future, all six cited lack of funding as a barrier to introduction and five cited lack of manpower (Figure 1f). Where prehabilitation was already provided, 11 units (52.4%) received full funding from the trust and in 6 centres (28.6%) the service relied on non-renewing funding. In two units (9.5%) the service was unfunded, three centres (9.5%) relied on research funding and one unit relied on a charity to fund the service (Figure 1g).

Almost all (20/21; 95%) units included aerobic training (AT) in their prehabilitation programme and in seven units (33%) that was the only component. AT was combined with resistance training in 11 (52%) units and with flexibility training in 4 (19%). Two units (10%) included sensorimotor training (Table 1).

**Table 1:** Details of current prehabilitation provision in England and Wales

Question		*n* (*N* = 21)	%
Who is principally responsible for delivering this service?	NHS physiotherapist	11	52
	Clinical exercise physiologist	2	10
	NHS specialist nurses	5	24
	Nutritionist/dietician	1	5
	Psychologist	1	5
	Anaesthetic team	1	5
	Community gyms and exercise specialists	1	5
When is this service delivered?	At diagnosis	9	43
	At diagnosis; during neoadjuvant therapy	1	5
	At diagnosis; during neoadjuvant therapy; before surgery	5	24
	During neoadjuvant therapy; before surgery	3	14
	During neoadjuvant therapy; before and after surgery	1	5
	During neoadjuvant therapy; before surgery; during palliative therapy	1	5
	During neoadjuvant therapy	1	5
What is the nature of the prehabilitation programme?	Aerobic training	7	33
	Aerobic training; resistance training	7	33
	Aerobic training; flexibility training	1	5
	Aerobic training; resistance training; flexibility training	1	5
	Aerobic training; resistance training; flexibility training; sensorimotor training	2	10
	Aerobic training; smoking cessation	1	5
	Aerobic training; resistance training; psychologist; nutritionist	1	5
How is baseline fitness measured prior to commencing the prehabilitation programme?	Not measured	5	24
	CPET	12	57
	6-Minute walk test	8	38
	Sit to stand test	9	43
	Chester step test	1	5
	Walk up 2 floors	1	5
	Hand grip strength	1	5
	Full assessment including mobility and medical fitness	1	5
How is response to prehabilitation programme objectively measured?	Not measured	9	43
	CPET	9	43
	6-minute walk test	4	19
	Sit to stand test	5	24
	HRQoL, WHODAS, ISWT	1	5
	Bespoke outcomes framework	1	5
	Hand grip strength	1	5

CPET = cardiopulmonary exercise test; HRQoL = Health-related quality of life; NHS = National Health Service; ISWT = incremental shuttle walk test; WHODAS = World Health Organization Disability Assessment Schedule.

Baseline fitness was measured by cardiopulmonary exercise testing (CPET) in 12 (57%) centres, although in two this was performed on a selective basis (Table 1). Five centres (24%) did not routinely measure baseline fitness. Eight units (38%) used 6-minute walk tests (6MWT) with nine units (43%) using a sit to stand test, with some utilising both (Table 1). Other baseline tests included hand grip dynamometry, the Chester step test and ‘walking up two flights of stairs’.

Fitness response to prehabilitation was not routinely measured in nine units (43%), whereas others used single or multiple validated assessments. CPET was used in nine units (43%) (one selectively). Five units (24%) repeated sit to stand test and four units (19%) repeated 6MWT. Two units assessed responses with a combination of quantitative and qualitative assessments as part of clinical trials (Table 1).

The majority (11/21; 52.4%) of prehabilitation programmes were delivered by NHS physiotherapists, and in NHS hospitals (11/21; 52.4%) (Table 1, Figure 1d). NHS specialist nurses were responsible for delivering the service in 5/21 (24%) centres. Two centres (10%) employed clinical exercise physiologists. One centre employed community exercise specialists in community gyms. Alongside the 11 centres that used NHS hospitals to deliver their service, 1 used an NHS facility outside the hospital and 4 (19%) used virtual, at-home programmes. Five (24%) used non-NHS facilities to deliver prehabilitation programmes (Figure 1d).

### What does an ideal prehabilitation service look like?

**Figure 2:**
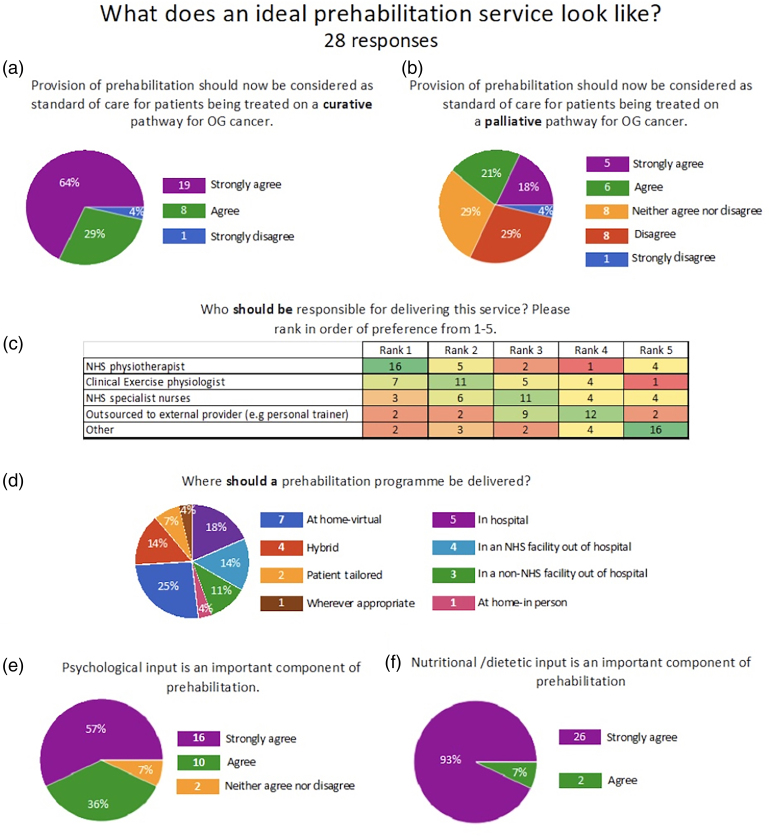
What does an ideal prehabilitation service look like? (a) Pie chart showing almost unanimous agreement that prehabilitation should be considered standard of care for those on a curative pathway. (b) Pie chart showing that many do not agree with prehabilitation for those on a palliative pathway. (c) Heatmap table of who respondents believe should be responsible for delivering prehabilitation, showing a clear preference for physiotherapists and physiologists. (d) Pie chart showing the lack of consensus on where prehabilitation should be delivered. (e,f) Pie charts show that almost all respondents believe that psychological and nutritional input are important features of a prehabilitation programme.

There was near-universal agreement that prehabilitation should be considered standard of care for OGC patients on a curative pathway (27/28; 96%) and 27 respondents strongly agreed with this statement (Figure 2a). In the palliative setting, 9 participants (32.1%) disagreed or strongly disagreed that prehabilitation should be the standard of care, whereas 11 respondents (39.3%) strongly agreed or agreed (Figure 2b). Most respondents believed that NHS physiotherapists (57%) or clinical exercise physiologists (25%) were best placed to provide the prehabilitation service. Only two respondents (7%) believed prehabilitation would be better outsourced to other providers and three (10.7%) believed that specialist nurses would be best placed to provide prehabilitation (Figure 2c).

The majority of respondents (27/28; 96%) believed that prehabilitation should begin from the time of diagnosis. Eleven respondents (39%) believed that prehabilitation should be delivered to patients being treated on a palliative pathway for OG cancer (Figure 2b).

There were a variety of opinions as to where prehabilitation programmes should be delivered (Figure 2d) and what outcomes should be measured. When asked whether prehabilitation should be included as a quality indicator in national audits such as NOGCA, the majority either agreed (*n* = 15; 53.6%) or strongly agreed (*n* = 8; 28.6%), whereas 4 (14%) respondents neither agreed nor disagreed and only 1 (4%) respondent disagreed.

Most (26/28; 93%) believed that psychological input was an important part of prehabilitation (Figure 2e) but only 12 units (43%) had access to a clinical psychologist for OGC patients. All respondents (28; 100%) believed that nutritional support was an important part of prehabilitation (Figure 2f).

We asked respondents to select those quantitative or qualitative outcomes that they felt could be improved by prehabilitation and those they thought were important to measure (Table 2).

**Table 2:** Variables that respondents believe prehabilitation could improve and those they thought it was important to measure

		*n* (*N* = 28)	%
What outcomes do you believe prehabilitation may improve?	Activity levels	28	100
	Smoking cessation	28	100
	Fitness	26	93
	Quality of life	26	93
	Likelihood of proceeding to surgery	25	89.3
	Complication rates after surgery	25	89.3
	Body composition	23	82.1
	Length of postoperative hospital stay	23	82.1
	Completion of neoadjuvant therapy	22	78.6
	Likelihood of receiving adjuvant treatment	21	75.0
	Overall survival	20	71.4
	Blood markers of inflammation and immunity	13	46.4
	Disease-free survival	11	39.3
	Tumour regression/response to chemotherapy	6	21.4
What outcomes are important to measure for patients on a prehabilitation programme?	Complication rates after surgery	26	93
	Length of postoperative hospital stay	26	93
	Quality of life	25	89.3
	Fitness	24	85.7
	Likelihood of proceeding to surgery	23	82.1
	Smoking cessation	21	75.0
	Completion of neoadjuvant therapy	21	75.0
	Likelihood of receiving adjuvant treatment	21	75.0
	Activity levels	20	71.4
	Overall survival	20	71.4
	Body composition	19	67.9
	Blood markers of inflammation and immunity	11	39.3
	Disease-free survival	10	35.7
	Tumour regression/response to chemotherapy	8	28.6

Respondents believed that prehabilitation could improve smoking cessation (28/28; 100%), activity levels (28/28; 100%), quality of life (26/28; 93%) fitness (26/28; 93%), surgical complication rates (25/28; 89.3%), likelihood of proceeding to surgery (25/28; 89.3%) and overall survival (20/28; 71.4%). Twenty-six of 28 respondents (93%) believed that length of stay, and complication rates should be measured and 25 (89.3%) believed that quality of life should be measured. Fitness (24/28; 85.7%) and likelihood of proceeding to surgery (23/28; 82.1%) were also cited by the majority of respondents.

## Discussion

Among respondents from this survey, including 28 OGC surgical centres in England and Wales, almost all participating surgeons believed that prehabilitation should be considered standard of care for patients undergoing OGC surgery. The majority believe that the ideal programme should be delivered from initial diagnosis and maintained until at least the time of surgery, and that the programme should consist of aerobic and resistance exercise training, delivered by NHS physiotherapists or clinical exercise physiologists. Nutrition support and psychological support should also be provided as part of a holistic multidisciplinary package, the latter potentially on a selective basis. Those surveyed believed that delivering this service would improve quality of life, reduce complications, reduce length of stay and may improve cancer survival. There were a variety of opinions on the exact type of exercise intervention or the location where that intervention should be delivered.

It has been demonstrated in multiple studies that delivering a prescribed exercise programme during chemotherapy is feasible and safe.^7,9,13,14^ OGC patients can potentially benefit from a neo-adjuvant period between diagnosis and surgery where adjuncts to traditional cancer treatments such as prescribed exercise, nutrition and psychological support can be delivered.^7,8,14,15^ Small, heterogeneous studies have shown improvements in objectively measured fitness and body composition as well as improved oncological outcomes associated with long-term disease-free survival.^7,8,16–20^ A meta-analysis of physiotherapy regimes, which consisted largely of inspiratory muscle training, showed an improvement in postoperative morbidity with the intervention.^21^

Studies so far have focused on repeating small-scale interventions with exercise programmes, baseline and response assessments and outcome measures that vary significantly from trial to trial.^7–9^ Therefore, there remains a lack of consensus regarding what exercise interventions should be delivered and very little evidence to support one intervention over another. Significant variations in practice were highlighted by the survey, for example, the use of baseline fitness assessment and response to intervention assessment. These principles have been laid out in recent societal guidance, although the exact modality for measuring fitness remains unclear, with variable use of CPET nationally.^22^ Whether such guidance becomes an accepted standard for accreditation or commissioning of services remains unclear. Similarly, the location of prehabilitation delivery varies significantly and certain aspects would have been interesting to explore in more detail, such as the different models of ‘in-house’ tumour-group-specific prehabilitation vs more generic larger scale, externally delivered programmes. In reality, the best solution for different centres may vary depending on a range of factors such as resource availability, patient demographics and geographical size. Currently, there is no conclusive evidence for the superiority of one type of programme over another, with numerous questions outstanding that may only be answered by dedicated prospective trials. It would seem that remote delivery of prehabilitation is safe and feasible, a change that was dictated by the Covid pandemic and led to the adaptation of randomised trial protocols (The Wessex Fit-4-Cancer Surgery Trial-WesFit; Safefit trial: virtual clinics to deliver a multimodal intervention to improve psychological and physical well-being in people with cancer).^23,24^ In the absence of conclusive evidence, centres have largely devised their own solutions, presumably based on an individualised assessment of need vs resources.

Within the survey there was strong support for expert dietitian involvement in multidisciplinary prehabilitation; however, the evidence supporting best practice in prehabilitation nutrition is hampered by the same small-scale heterogeneous studies that currently inform exercise interventions.^25,26^

The individuals surveyed in this study were taken from a contact list held by NOGCA and represented centres performing the majority of the volume of cases performed in England and Wales. Surgeons are only one part of a multidisciplinary team delivering prehabilitation programmes. The questionnaire specifically asked for the most appropriate individual at the institution to respond (this did not necessarily have to be the individual in receipt of the request) and to respond on behalf of the unit. The first part of the survey included largely factual statements regarding current provision of prehabilitation, which should not have varied greatly regardless of who completed the questionnaire. The second part of the survey canvassed surgeons’ opinions and excludes the valuable knowledge of dieticians, physiotherapists, exercise specialists, oncologists, anaesthetists and psychologists. Such multidisciplinary initiatives have recently concluded with societal support. The survey did not consider the economic benefits that may be ascribed to prehabilitation; however, a reduction in complication rates and length of stay, which respondents believed could be improved by prehabilitation, might be expected to influence the overall cost to funders.

Successful prehabilitation depends upon adequate adherence to the programme. A systematic review reported prehabilitation adherence rates of 70% and a further study showed that participants dropping out of a prehabilitation trial had worse survival than both the intervention and control groups.^5,27^ Patient participation remains a major obstacle to effective prehabilitation, especially when dealing with elderly, comorbid OGC patients. When determining the services to be offered, it is important to consider not only the optimal prehabilitation programme in principle, but also what can be realistically achieved with high levels of compliance in the OGC patient population.

## Conclusion

OGC surgical units appear to be convinced of the value of prehabilitation and believe it should be already considered standard of care. The survey presented suggests that there is an appetite for higher quality evidence, guidelines and agreed standards to secure the resources needed to deliver these services. Given the conspicuous gap between evidence base and consensus opinion, a large multicentre trial is required, alongside societal guidelines, to reduce the heterogeneity that currently exists. While awaiting the results of these studies, further small-scale feasibility trials are unlikely to convince funders of the value of investing in prehabilitation.^23,24^ If the predicted benefits in terms of treatment completion rates, complication rates and oncological outcomes can be clearly demonstrated then efforts to optimise specific aspects of the intervention can be refined over time.
